# Influence of Fractionation and Beam Sequencing on Absorbed Dose to Circulating Lymphocytes During Ultra-High Dose Rate and Conventional Radiotherapy: An In Silico Study

**DOI:** 10.1200/CCI-26-00091

**Published:** 2026-07-17

**Authors:** François de Kermenguy, Camilla Satragno, Mohammed El-Aichi, Ibrahima Diallo, Cristina Veres, Fereshteh Talebi, Cathyanne Schott, Eric Deutsch, Charlotte Robert

**Affiliations:** ^1^ Inserm U1030 Radiothérapie Moléculaire et Innovation Thérapeutique, Gustave Roussy, Université Paris-Saclay, Villejuif, France; ^2^ Department of Radiation Oncology, Mass General Brigham Cancer Institute and Harvard Medical School, Boston, MA; ^3^ Département de radiothérapie, Gustave Roussy, Villejuif, France

## Abstract

**PURPOSE:**

Several in silico models concluded that ultra-high dose rate (UHDR) radiotherapy could spare large quantities of circulating lymphocytes. However, preclinical studies failed to show a reduction in radiation-induced lymphopenia after UHDR irradiation compared with conventional dose rates (CONV). This study aims to investigate the influence of fractionation and beam sequencing on absorbed dose to circulating lymphocytes during CONV and UHDR irradiations.

**MATERIALS AND METHODS:**

The LymphoDose framework was applied to a cohort of 162 patients treated for brain tumors with 3D conformal radiotherapy. Four scenarios of UHDR treatment were compared: (S1) one fraction with all beams delivered simultaneously, (S2) one fraction with sequentially delivered beams, (S3) three fractions with all beams delivered simultaneously, and (S4) three fractions with sequentially delivered beams.

**RESULTS:**

UHDR fractionation and the beam delivery scheme had a significant impact on irradiated blood volume (1.3% ± 0.1% for S1 *v* 13% ± 3.2% for S4) and lymphocyte pool (12.5% ± 0.1% for S1 *v* 32.8% ± 0.2% for S4). UHDR scenarios primarily irradiate lymphocytes through exposure of head-and-neck lymph node rather than circulating blood. CONV and UHDR result in distinct temporal dose patterns for lymphocytes, characterized by either numerous low-dose fractions or a few high-dose pulses.

**CONCLUSION:**

The doses delivered to lymphoid organs account for a substantial portion of the total dose received by the lymphocyte pool, showing only a limited difference between UHDR and CONV in terms of lymphocyte exposure. The fractionation strategy of UHDR irradiation beams could play an important role in successfully translating UHDR treatments into clinical practice.

## Graphical abstract

**Figure:**
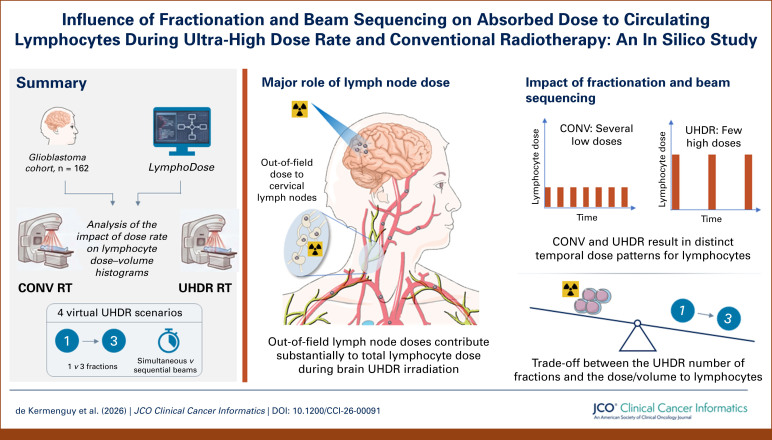

## INTRODUCTION

Ultra-high dose rate (UHDR) radiotherapy is attracting growing interest due to its differential effect of reducing toxicities in healthy tissue while maintaining tumor control.^1-3^ However, the radiobiological mechanisms of the FLASH effect remain poorly understood. One of the hypotheses was that, compared with conventional dose rate (CONV) irradiations, the shorter irradiation times associated with UHDR could spare a significant proportion of circulating immune cells, including lymphocytes.^4-6^

**CONTEXT**

**Key Objective**
What is the impact of fractionation and beam sequencing on the absorbed dose to blood and circulating lymphocytes during ultra-high dose rate (UHDR) brain irradiations?
**Knowledge Generated**
The doses delivered to lymphoid organs account for a substantial portion of the total dose received by the lymphocyte pool, showing a limited difference between UHDR and conventional dose rates in terms of lymphocyte exposure. The fractionation strategy of UHDR irradiation should be carefully considered to successfully translate UHDR treatments into clinical practice.
**Relevance *(R.C. Rockne)***
This research demonstrates how computational approaches can be used to improve radiation therapy by reducing the dose to immune cells circulating in target organs. The work advances precision medicine in radiation oncology by leveraging clinical data and computational modeling to personalize radiation dose.*
*Relevance section written by *JCO CCI* Associate Editor Russell C. Rockne, PhD.

In this context, several numerical simulations have been developed to estimate the dose received by the blood lymphocytes during UHDR irradiations.^6,7^ Using a simple compartmental model, Jin et al^4^ showed a reduction in the killed fraction of circulating immune cells from 90% to 100% at CONV to 5%-10% at 40 Gy/s UHDR irradiation. Mimicking abdominal 400 Gy/s UHDR irradiation in mice with doses of 10-40 Gy, Cucinotta and Smirnova^8-10^ showed that the rate of surviving lymphocytes increased with the dose rate up to a limit proportional to the volume of blood irradiated in the field. Using a detailed brain vasculature model, Galts and Hammi^11^ showed that proton beam scanning UHDR irradiation reduced the depletion rate of circulating lymphocytes by 69.2% compared with conventional fractionated intensity-modulated proton therapy. Recently, Li et al^12^ leveraged an enhanced version of HEDOS^13^ to show that lymphocyte levels 3 months after treatment were 30% higher with UHDR compared with CONV in a cohort of 17 patients with head and neck cancer.

However, several preclinical studies have failed to show such a sparing effect. Venkatesulu et al^14^ observed longer and more severe lymphocyte depletion during mice cardiac and splenic 35 Gy/s UHDR irradiation compared with CONV irradiation. Iturri et al^15^ found no significant differences in circulating immune populations between UHDR (257 ± 2 Gy/s) and CONV proton irradiation delivered at a single 25 Gy fraction to the rat brain. By investigating CONV and UHDR immune response in various murine tumor models, Almeida et al^16^ explicitly contradicted the immune-sparing hypothesis of FLASH effect. Saenz et al^17,18^ only observed a low and nonsignificant difference in blood circulating lymphocytes in mice after FLASH compared with CONV. They, however, reported a FLASH sparing of naïve and activated lymphocytes in tumor-draining lymph nodes (LNs). Recently, Tao et al^19^ showed that single (17 Gy) and multifraction (2 Gy×5) thoracic FLASH irradiations in mice improve lymphopenia recovery relative to CONV, despite showing only minimal differences in peripheral lymphocyte levels immediately after treatment.

This study aims to explore the discrepancies between in silico predictions and preclinical observations, from a dosimetric point of view. Although many previous publications used peripheral blood dose as a proxy for circulating lymphocyte doses, LymphoDose considers the complex recirculation dynamics of lymphocytes and the dose to distant LN stations. We therefore investigated the dosimetric impact of varying UHDR fractionation schemes and beam delivery sequencing on both blood and lymphocyte exposure in patients treated for brain tumors.

## MATERIALS AND METHODS

### Patient Selection

Numerous UHDR photon irradiator designs have been proposed in the literature.^20-25^ The majority of approaches involve delivering a limited number of static sequential beams through gantry or subject rotation. Only the pluridirectional high-energy agile scanning electronic radiotherapy (PHASER)^20^ proposed to deliver ultra-rapid highly conformal image-guided radiation therapy from 16 stationary UHDR beams. To approach treatment plans produced by these types of photon irradiators, only 3D conformal radiotherapy (3DCRT) treatments were chosen, as they involve a limited number of static irradiation beams. We thus retrospectively identified patients with high-grade glioma from an institutional cohort with the following inclusion criteria: (1) treatment at Gustave Roussy, Villejuif, France, between 2009 and 2021, (2) a pathological and/or molecular diagnosis of high-grade glioma, including grade 4 astrocytoma isocitrate dehydrogenase (IDH)-mutant and IDH-wild-type glioblastoma,^26^ (3) planned treatment with 3DCRT, and (4) availability of dosimetric data (planning CT, DICOM RTSS, DICOM RTDose, and DICOM RTPlan).

### UHDR Treatment Plan

The FLASH effect is generally observed in preclinical settings at a total dose D>8 Gy in a single fraction and from a single beam angle.^2,27^ However, for a clinical translation, these conditions need to be adapted to maintain good dose conformity for larger volumes and to broaden the therapeutic window. It is therefore necessary to divide the treatment into several fractions (usually three or more) and to use multiple beam directions at each fraction.^2^ Four UHDR irradiation scenarios were therefore considered:

- (S1) One fraction ($N_{F,UHDR}=1$), with all beams delivered simultaneously,
- (S2) One fraction ($N_{F,UHDR}=1$), with beams delivered sequentially ($\Delta t_{B}=10\;s$),
- (S3) Three fractions ($N_{F,UHDR}=3$), with all beams delivered simultaneously, and
- (S4) Three fractions ($N_{F,UHDR}=3$) with beams delivered sequentially ($\Delta t_{B}=10\;s$).

Scenarios S1 and S3 were inspired by PHASER-like machines, capable of delivering all irradiation beams nearly simultaneously from different angles.^20^ Scenarios S2 and S4 were based on more conventional irradiation schemes, with a gantry rotation time of $\Delta t_{B}=10\;s$ between successive irradiation beams.

The number of UHDR beams $N_{B,UHDR}$ was defined as equal to the number of beams of the corresponding patient CONV irradiation $N_{B,CONV}$, during the actual patient treatment. The UHDR delivery dose rate was fixed to 100 Gy/s^2^ to the planning treatment volume (PTV). The UHDR dose per fraction $d_{UHDR}$ was calculated to achieve the same tumor biological equivalent dose^28^ as CONV. Denoting by α and β the tumor cell radiosensitivity parameters and by $d_{CONV}$ and $N_{F,CONV}$ the dose per fraction and the number of fractions delivered during CONV treatment, respectively, the corresponding UHDR dose per fraction $d_{F,UHDR}$ was defined as the solution to the following equation:

$$BED_{UHDR}=BED_{CONV}⟺\;\left(N_{F,UHDR}\cdot d_{UHDR}\right)\cdot \left(1+\frac{d_{UHDR}}{\alpha /\beta }\right)=\left(N_{F,CONV}\cdot d_{CONV}\right)\cdot \left(1+\frac{d_{CONV}}{\alpha /\beta }\right)\;⟺\;\frac{N_{F,UHDR}}{\alpha /\beta }\cdot d_{UHDR}^{2}+N_{F,UHDR}\cdot d_{UHDR}-\left(N_{F,CONV}\cdot d_{CONV}\right)\cdot \left(1+\frac{d_{CONV}}{\alpha /\beta }\right)=0$$

This is a polynomial of degree 2 of parameters $a=\frac{N_{F,UHDR}}{\alpha /\beta }$, $b=N_{F,UHDR}$, and $c=-\left(N_{F,CONV}\cdot d_{CONV}\right)\cdot \left(1+\frac{d_{CONV}}{\alpha /\beta }\right)$, with only one real and positive root. We assumed α/β=8 Gy for the tumor.^29^ The total dose delivered by the UHDR treatment was $D_{UHDR}=N_{F,UHDR}\cdot d_{F,UHDR}$. For scenarios S1 and S2, $N_{F,UHDR}=1$, thus $D_{UHDR}=d_{UHDR}$. In simultaneous-beam scenarios S1 and S3, the whole-treatment CONV dose map was multiplied by a scaling factor $\delta =\frac{D_{UHDR}}{D_{CONV}}$. In sequential-beam scenarios S2 and S4, each beam CONV dose map was multiplied by δ.

### LymphoDose Application

LymphoDose was applied to each patient, taking into account the 3DCRT CONV and the four virtual UHDR treatments. LymphoDose^30^ is an in silico framework based on a kinetic Monte Carlo algorithm. It consists of two interconnected compartment models, which describe the slow recirculation of lymphocytes between lymphoid organs and their rapid circulation in the bloodstream. For each case, both the dose to peripheral blood and the dose to circulating lymphocytes, taking into account recirculation/homing between lymphoid organs and the dose received by LNs in the head-and-neck (HN) region, were computed. To do so, the following steps were applied to each patient. Dose maps for each irradiation beam were retrieved from the treatment planning system DOSIsoft IsoGray (Paris, France). These maps had previously been calculated using a Clarkson-type analytic dose calculation method. Right and left LN areas, including Ia, Ib, II, III, IVa, V, VIIa, and VIIb regions,^31^ were automatically contoured using ART-Plan software v1.11.5 (TheraPanacea, Paris, France). The extended out-of-field dose maps from CONV treatments were generated with a previously used in-house deep learning neural network.^30,32^ This step was necessary to compute doses to LNs located at a distance from the irradiation field in brain tumors. Whole-brain dose volume histogram (DVH) and volume-weighted mean doses to HN LN ($\bar{OOF_{CONV}}$) were then computed using dicompyler-core version 0.5.6.^33^ UHDR mean doses to HN LN were equal to $\bar{OOF_{UHDR}}=\delta \cdot \bar{OOF_{CONV}}$. LymphoDose simulations were performed considering $10^{4}$ particles for blood doses and $10^{5}$ for lymphocytes doses. Paired nonparametric statistical analyses were used to compare DVH-extracted mean doses to blood and circulating lymphocytes across all treatment scenarios (CONV, S1, S2, S3 and S4). Overall differences across the five scenarios were first assessed using the Friedman test. Pairwise post hoc comparisons were then performed using Wilcoxon signed-rank tests. *P* values were adjusted for multiple comparisons using the Holm-Bonferroni method. For each pairwise comparison, the paired median difference, its 95% bootstrap CI, and the effect size were reported.

### Ethics Approval and Consent to Participate

The utilization of this retrospective training cohort was performed in accordance with the General Data Protection Regulation and approved by the local ethical committee (institutional review board number 2023-298).

## RESULTS

### Cohort Characteristics and Dose Delivery in CONV Treatments

A total of 162 patients were included in this analysis (Table 1). The mean dose prescribed was $D_{CONV}=58.7\;\pm \;4.9$ Gy with a mean number of fractions $N_{F,CONV}=29\;\pm \;3.7$. Each fraction was composed of an average of $N_{B}=3.9\;\pm \;1.0$ beams, each lasting on average $t_{B,CONV}=22.5\;\pm \;14.0$ s. The average dose to LN with CONV was $\bar{OOF_{CONV}}=4.3\;\pm \;1.5$ Gy.

**TABLE 1.** Description of the Cohort of 162 Patients With Glioblastoma Treated With CONV Irradiation

Characteristic	No. (%) or Mean ± SD/Median (range)
Age, years	57.1 ± 13.9/59 (18.3-83.9)
Sex (male)	118 (73)
Tumor location	
Temporal	51 (31.5)
Frontal	46 (28.4)
Parietal	25 (15.4)
Others	60 (24.7)
PTV volume, cm^3^	295.2 ± 149.9/294 (4-637)
Prescribed dose $D_{CONV}$, Gy	58.7 ± 4.9/60 (33-60)
Dose per fraction $d_{CONV}$, Gy	2.0 ± 0.2/2.0 (1.8-3.3)
Number of fractions $N_{F,CONV}$	29.0 ± 3.7/30 (10-33)
Number of beams per fraction $N_{B,CONV}$	3.9 ± 1.0/4 (2-8)
Interbeam duration $\Delta t_{B}$, s	10
Beam duration $t_{B,CONV}$, s	22.5 ± 14.0/17.7 (5.6-75.5)
Mean head-and-neck lymph node dose $\bar{OOF_{CONV}}$, Gy	4.3 ± 1.5/3.8 (2.6-10.7)

Abbreviations: CONV, conventional dose rates; PTV, planning treatment volume; SD, standard deviation.^6^

### Virtual UHDR Treatments

For the single-fraction scenarios (S1 and S2), virtual UHDR treatments (Table 2) delivered a mean dose of $D_{CONV}=20.6\;\pm \;0.9$ Gy to the PTV and a mean HN LN dose $\bar{OOF_{UHDR}}\;\text{of}\;1.5\;\pm \;0.5$ Gy. When three fractions were considered (S3 and S4 scenarios), virtual UHDR treatments (Table 2) delivered a mean dose of $D_{CONV}=31.7\;\pm \;1.5$ Gy and a mean HN LN dose $\bar{OOF_{UHDR}}\;\text{of}\;2.3\;\pm \;0.8$ Gy. In every case, one fraction delivered an average of $N_{B}=3.9\;\pm \;1$ beams, with an average beam delivery time $t_{B,UHDR}$ of 0.21 ± 0.01 s, $5.7\cdot 10^{-2}\pm \;1.6\cdot 10^{-2}\;s$, 0.11 ± 0.01 s, and $2.9\cdot 10^{-2}\pm \;0.8\cdot 10^{-2}$ s for S1, S2, S3, and S4 respectively.

**TABLE 2.** Description of the Four UHDR Treatment Scenarios Simulated in the Cohort of 162 Patients With Glioblastoma

Characteristic	S1	S2	S3	S4
Prescribed dose $D_{UHDR}$, Gy	20.6 ± 0.9/20.8 (15.7-20.8)		31.7 ± 1.5/32.1 (23.5-32.1)	
Dose per fraction $d_{UHDR}$, Gy	20.6 ± 0.9/20.8 (15.7-20.8)		10.6 ± 0.5/10.7 (7.8-10.7)	
Number of fractions $N_{F,UHDR}$	1		3	
Number of beams per fraction $N_{B,UHDR}$	3.9 ± 1.0/4 (2-8)			
Interbeam duration $\Delta t_{B}$, s	All beams delivered simultaneously	10	All beams delivered simultaneously	10
Beam duration $t_{B,UHDR}$, s	0.21 ± 0.01/0.21 (0.16-0.21)	$5.7\cdot 10^{-2}\pm 1.6\cdot 10^{-2}$/$5.7\cdot 10^{-2}$ ($2.6\cdot 10^{-2}$-$10.4\cdot 10^{-2}$)	0.11 ± 0.01/0.11 (0.08−0.11)	$2.9\cdot 10^{-2}\pm 0.8\cdot 10^{-2}$/$2.7\cdot 10^{-2}$ ($1.3\cdot 10^{-2}$-$5.3\cdot 10^{-2}$)
Mean head-and-neck lymph node dose $\bar{OOF_{UHDR}}$, Gy	1.5 ± 0.5/1.3 (0.9-3.7)		2.3 ± 0.8/2 (1.4-5.7)	

Abbreviation: UHDR, ultra-high dose rate.

### LymphoDose Results

**FIG 1.**
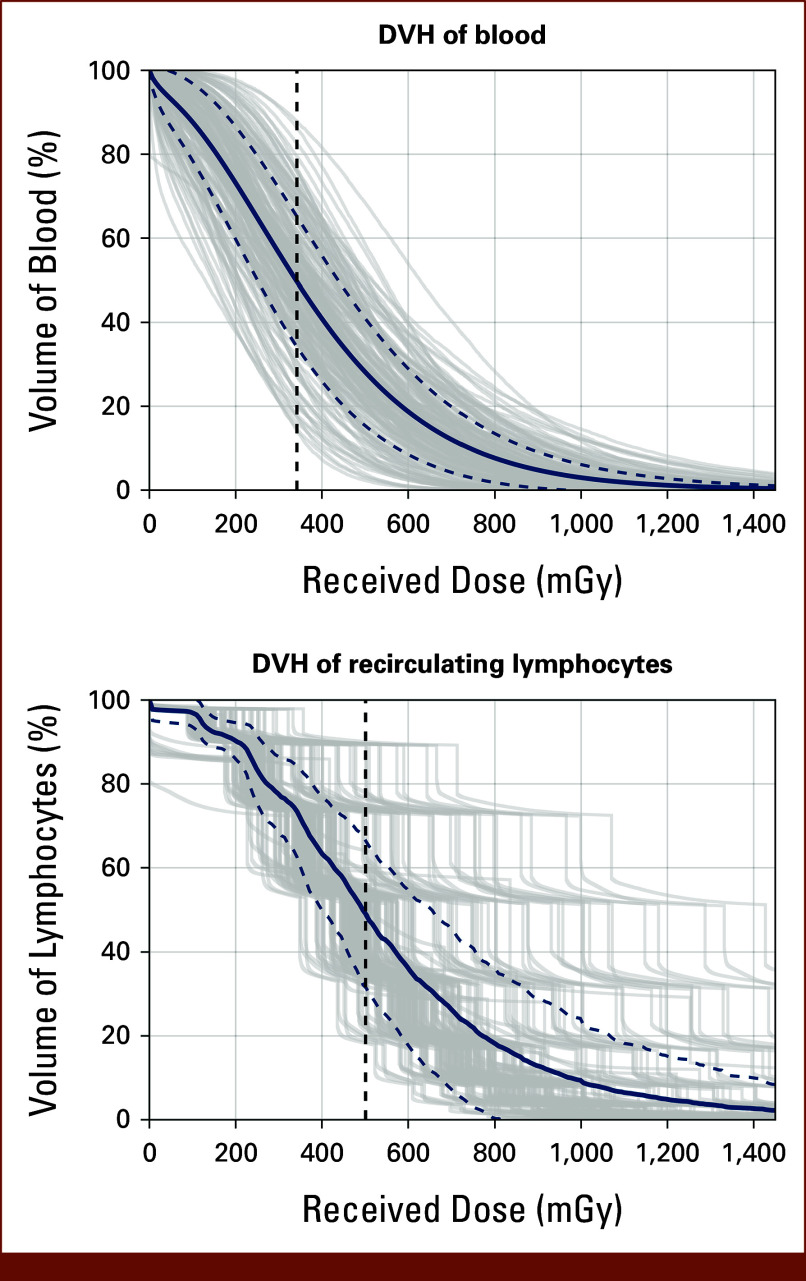
DVH of blood (top) and circulating lymphocytes (bottom) at the end of the CONV treatment. In each figure, the mean DVH over the whole cohort is shown in solid blue, the associated standard deviation in dotted blue line, the DVH for each patient in the cohort in solid light gray, and the D50% as a vertical dotted black line. CONV, conventional dose rates; DVH, dose volume histogram.

For CONV irradiations, the mean dose to irradiated volume $V_{>\;0\;\text{Gy}\;}$ was 0.39 ± 0.1 Gy and 0.57 ± 0.18 Gy for peripheral blood and circulating lymphocytes, respectively (Fig 1 and Table 3). By the end of the treatment, nearly the entire blood volume had been irradiated ($V_{>0\;\text{Gy}\;}=99.3\%\;\pm \;1.8\%$), as was nearly the entire lymphocyte pool, considering the HN LN dose ($V_{>0\;\text{Gy}}=98\%\;\pm \;2.5\%$).

**TABLE 3.** Different Metrics Summarizing the Irradiated Volumes and the Doses Received by the Blood and the Recirculating Lymphocyte Pool During CONV and UHDR (S1, S2, S3 and S4 irradiation scenarios) Treatments, Averaged Over the Whole Cohort

Model	Results	CONV	UHDR S1	UHDR S2	UHDR S3	UHDR S4
Blood dose	*V*_>0 Gy_, %	99.3 ± 1.8	1.3 ± 0.1	4.6 ± 1.2	3.7 ± 0.2	13.0 ± 3.2
	*V*_>0.125 Gy_, %	84.5 ± 10.4	1.3 ± 0.1	4.1 ± 1	3.6 ± 0.2	10.2 ± 2.6
	Mean dose, Gy	0.4 ± 0.1	11.2 ± 2.7	3.3 ± 1	5.9 ± 1.4	1.8 ± 0.5
	D50%, Gy	0.3 ± 0.1	9.7 ± 4.8	2.4 ± 1.6	5.2 ± 2.5	1.3 ± 0.8
	D2%, Gy	1.0 ± 0.2	22.9 ± 1.2	9.9 ± 2.1	11.6 ± 0.6	5.3 ± 1.1
Lymphocytes dose	*V*_>0 Gy_, %	98.0 ± 2.5	12.5 ± 0.1	12.7 ± 0.1	32.3 ± 0.1	32.8 ± 0.2
	*V*_>0.125 Gy_, %	94.3 ± 4	12.5 ± 0.1	12.7 ± 0.1	32.3 ± 0.1	32.7 ± 0.2
	Mean dose, Gy	0.6 ± 0.2	1.6 ± 0.5	1.5 ± 0.5	0.9 ± 0.3	0.9 ± 0.3
	D50%, Gy	0.6 ± 0.2	1.5 ± 0.5	1.5 ± 0.5	0.8 ± 0.3	0.8 ± 0.3
	D2%, Gy	1.2 ± 0.4	1.5 ± 0.5	1.6 ± 0.6	1.5 ± 0.5	1.8 ± 0.5

$\text{NOTE}.\;V_{>0\;\text{Gy}}$: volume of lymphocytes having received a non-null dose, $V_{>0.125\;\text{Gy}}$: volume of lymphocytes having received a dose greater or equal to 0.125 Gy. The average, median and 98th percentile doses are reported. The last three metrics are calculated on irradiated blood/lymphocytes only ($V_{>0\;\text{Gy}}$).^6^

Abbreviations: CONV, conventional dose rates; UHDR, ultra-high dose rate.

**FIG 2.**
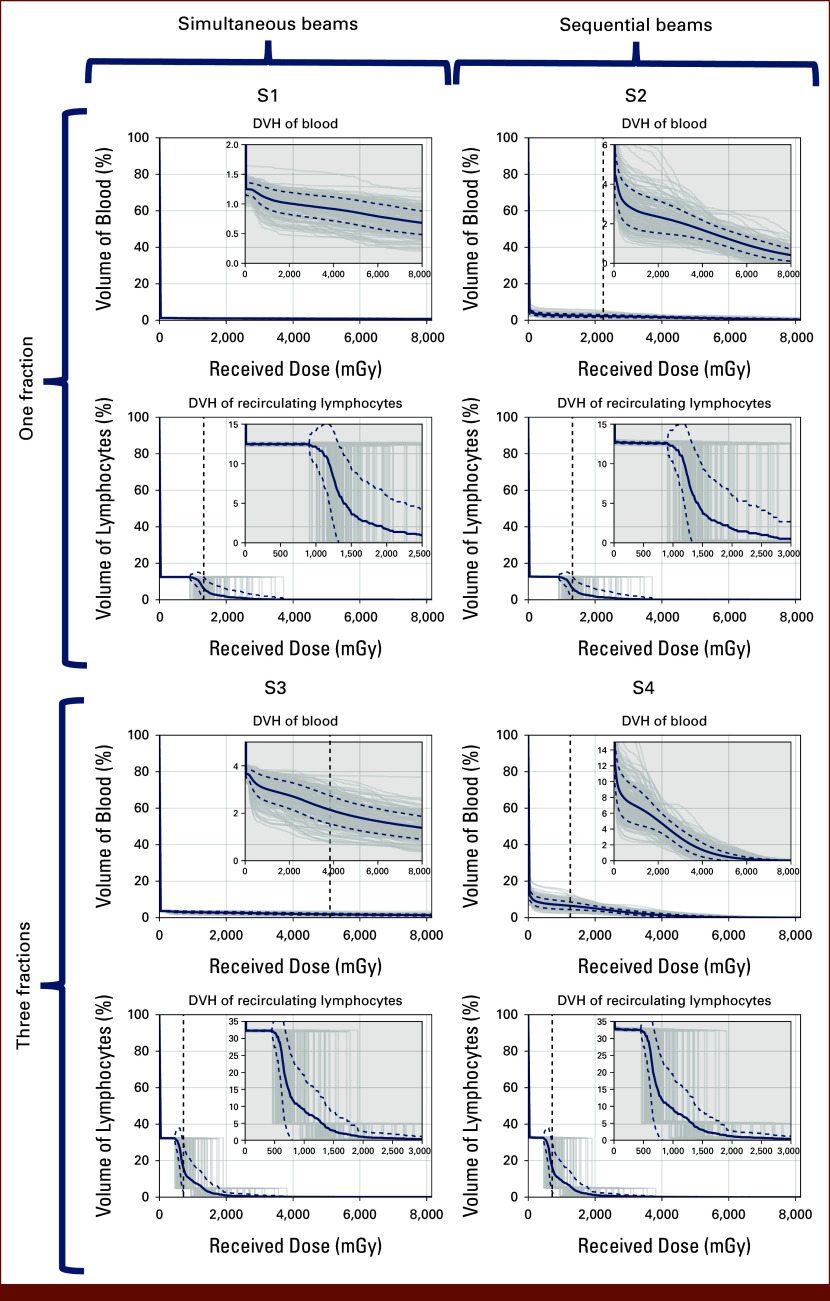
DVH of blood (top) and circulating lymphocytes (bottom) at the end of UHDR treatment for S1 (top, left), S2 (top, right), S3 (bottom, left), and S4 (bottom, right) scenarios. S1: one fraction with simultaneous beam delivery; S2: one fraction with sequential beams; S3: three fractions with simultaneous beam; S4: three fractions with sequential beams. In each figure, the mean DVH over the whole cohort is shown in solid blue, the associated standard deviation in dotted blue line, the DVH for each patient in the cohort in solid light gray, and the D50% as a vertical dotted black line. A zoom subplot is also shown. DVH, dose volume histogram; UHDR, ultra-high dose rate.

For UHDR irradiations (Fig 2 and Table 3), the blood irradiated fraction $V_{>\;0\;\text{Gy}\;}$ ranged from 1.26% ± 0.11% (at a mean dose of 11.24 ± 2.65 Gy) to 12.98% ± 3.21% (at a mean dose of 1.76 ± 0.50 Gy) for scenarios S1 and S4, respectively. On average, one third of circulating lymphocytes were irradiated in the two 3-fraction scenarios (S3 and S4), at an average dose of about 1 Gy. The monofractionated scenarios (S1 and S2) irradiated only an eighth of the lymphocytes, but at higher doses of about 1.6 Gy.

Overall paired comparisons across the five irradiation scenarios demonstrated highly significant differences for both the mean blood dose and the mean circulating lymphocyte dose (Friedman test: $\chi^{2}=646.4,p<\;1.39\times 10^{-6}\;\text{and}\;\chi^{2}=638.8,p\;<\;6.30\times 10^{-6}$, respectively) (Data Supplement). For blood dose, all pairwise scenario comparisons remained statistically significant after correction (all adjusted $P\;\text{values}\;<2.5\times 10^{-6}$), with consistently very large effect sizes (rank-biserial correlation coefficients close to ±1). For circulating lymphocyte dose, all pairwise comparisons remained statistically significant after Holm correction. However, the median paired differences associated with beam sequencing comparisons remained markedly smaller than those associated with fractionation changes. For example, the median paired difference between S1 and S2 was −0.024 Gy (95% CI [−0.026 to−0.023]), with a smaller absolute dose variation despite a large standardized paired effect size. In contrast, comparisons involving different fractionation schemes showed substantially larger differences (eg, S1 *v* S3: median difference, −0.573 Gy,95% CI [−0.584 to−0.551]), also associated with very large effect sizes.

## DISCUSSION

LymphoDose was applied to a cohort of 162 patients with high-grade gliomas.^6^ Either CONV 3DCRT or four UHDR virtual photon irradiation scenarios were modeled. In line with previous simulations published in the literature, our results showed that UHDR irradiation significantly reduces the volume of blood directly irradiated in the field compared with CONV treatment (99.3% ± 1.8% in CONV *v* 13.0% ± 3.2% in the worst UHDR S4 scenario). However, these results are nuanced when we consider the dose to circulating lymphocytes, taking into account recirculation/homing between lymphoid organs and the dose received by HN LN. Even with UHDR, one eighth to one third of the lymphocyte pool was irradiated, at a high average dose ranging from 0.9 to 1.6 Gy, mainly via the dose received by the LN in the HN region.

The absorbed dose delivered to HN LN during UHDR was, on average, lower than that in CONV (1.5 ± 0.5 Gy for S1/S2 or 2.3 ± 0.8 Gy for S3/S4 compared with 4.3 ± 1.5 Gy) (Tables 1 and 2). This difference is attributed to the lower total dose administered in hypofractionated UHDR compared with CONV (20.6 ± 0.9 Gy in a single UHDR fraction or 31.7 ± 1.5 Gy across three UHDR fractions *v* 58.7 ± 4.9 Gy in CONV). However, the timing of delivery of these doses was very different between the two treatments: 2.3 ± 0.8 Gy were delivered in three UHDR fractions with S3 and S4 scenarios (ie, 0.8 ± 0.3 Gy per fraction) compared with 4.3 ± 1.5 Gy delivered in 29 ± 3.7 CONV fractions (ie, 0.2 ± 0.1 Gy per fraction). Knowing that in vivo T lymphocytes have an estimated lethal dose of 50% between 1 and 2 Gy,^34^ UHDR irradiations could induce more lethal damages to the irradiated pool than CONV, as large doses are delivered in short time frames, allowing minimal opportunity for cellular repair mechanisms to act. It should also be noted that these doses are probably too low to benefit from the FLASH sparing effect. Comparing ex vivo CONV (0.1 Gy/s) and UHDR (2,000 Gy/s) irradiation of peripheral blood lymphocytes, Cooper et al^35^ showed no significant difference in terms of DNA damage following single-dose irradiation of up to 20 Gy. It is therefore crucial to explicitly distinguish the impact of fractionation: although CONV exposes LNs to high cumulative doses distributed across numerous sublethal fractions allowing for repair, UHDR delivers lower absolute doses in single pulses that likely exceed the immediate apoptotic threshold of lymphocytes. Consequently, the experimentally observed absence of lymphocyte sparing does not necessarily contradict the existence of a FLASH effect but rather suggests that it is conditional and potentially inoperative for circulating or nodal lymphocytes at doses in the range of 1-2 Gy.

It is important to examine the impact of UHDR fractionation schemes and beam sequencing on the doses received by blood and the lymphocyte pool. For peripheral blood, the statistical analysis suggested that both fractionation and beam sequencing are important. Thus, to maximize blood sparing while maintaining acceptable tumoral conformation, a choice must be made between fractionation and number of beams per fraction. If the FLASH effect really does compensate for normal tissue toxicity, an interesting solution could be to deliver a different radiation beam at each daily fraction. However, the hypothesis of blood volume as a critical radiolytic target remains to be validated experimentally.^36^ For lymphocytes, the statistical analysis suggested that fractionation exerts the dominant effect, whereas beam sequencing effects, although statistically detectable, remain comparatively limited in magnitude.

Our study has several limitations. Although this work is primarily dosimetric, radiation-induced lymphopenia is likely driven by mechanisms more complex than purely cytotoxic effects of irradiation. Previous simulation work^7,13,30^ has shown that direct irradiation-induced lethality alone cannot satisfactorily explain the magnitude of lymphocyte depletion observed after CONV irradiations. This raises the possibility that UHDR irradiation, like CONV, may also trigger systemic immunomodulatory processes, such as the expansion of myeloid-derived suppressor cells^37^ or impaired T-cell recirculation.^38^ Some studies suggest potentially specific UHDR immune effects,^39^ such as macrophage reprogramming^40^ or Chk1-STAT3 axis modulation,^19^ but the current evidence is still sparse. Altogether, more experimental data along with truly mechanistic modeling are required to determine to what extent the distinct DVHs characterizing UHDR irradiation translate into meaningful biological differences in the development of lymphopenia.^7^ Then, the simulated UHDR dose maps probably lack realism, particularly as there is currently a limited number of device capable of delivering UHDR photon beams.^20-24^ Although using very high-energy electron (VHEE) or proton dose maps would be more realistic, vascular and lymphatic anatomy remains constant regardless of the particle type or dosimetry used. Even with protons or VHEE, cervical LNs are often exposed to exit or scatter doses, and 3DCRT can be considered as a worst-case scenario: the absence of clear lymphocyte sparing under these conditions primarily underscores the critical importance of dose confinement in clinical practice. Then, interpatient cerebrovascular variability can lead to uncertainties of up to 18% in estimated peripheral blood doses during cerebral irradiation^41^ and could be considered to improve the precision of results. An open question remains regarding the preservation of the FLASH effect in the context of beam splitting or fractionation. Recent work, including the study by Mascia et al^42^ and Sørensen et al,^43^ suggests that pauses between beam deliveries may compromise the maintenance of the FLASH effect, although definitive evidence is still lacking.

In conclusion, by applying the LymphoDose framework to a cohort of 162 patients with high-grade glioma, this study demonstrated that UHDR delivery does not inherently equate to dosimetric lymphocyte sparing. Going beyond the isolated blood dose evaluated in previous studies by considering the dose to lymphoid organs in addition to lymphocyte dynamics, we showed that a high fraction of the recirculating lymphocyte pool remains exposed to doses exceeding 1 Gy when undergoing UHDR treatment. In particular, UHDR number of fractions may matter more than dose rate per se when it comes to lymphocyte sparing. Irradiation schemes, varying in terms of number of fractions and beams, could have very different effects in terms of the dose received by the blood and lymphocytes. UHDR clinical translation should prioritize lymphoid-organ avoidance and reduced fractionation rather than relying solely on ultra-short delivery times.

## Data Availability

A data sharing statement provided by the authors is available with this article at DOI https://doi.org/10.1200/CCI-26-00091. Data necessary to interpret, verify and extend the research in the article are available from the corresponding authors upon request.
